# Percutaneous CT Fluoroscopy-Guided Core Needle Biopsy of Mediastinal Masses: Technical Outcome and Complications of 155 Procedures during a 10-Year Period

**DOI:** 10.3390/diagnostics11050781

**Published:** 2021-04-26

**Authors:** Caroline Burgard, Robert Stahl, Giovanna Negrao de Figueiredo, Julien Dinkel, Thomas Liebig, Dania Cioni, Emanuele Neri, Christoph G. Trumm

**Affiliations:** 1 Department of Nuclear Medicine, University Hospital, LMU Munich, 81377 Munich, Germany; 2 Institute for Diagnostic and Interventional Neuroradiology, University Hospital, LMU Munich, 81377 Munich, Germany; Robert.Stahl@med.uni-muenchen.de (R.S.); Thomas.Liebig@med.uni-muenchen.de (T.L.); christoph.trumm@med.uni-muenchen.de (C.G.T.); 3 Department of Radiology, University Hospital, LMU Munich, 81377 Munich, Germany; Giovanna.Negrao_de_Figueiredo@med.uni-muenchen.de (G.N.d.F.); Julien.Dinkel@med.uni-muenchen.de (J.D.); 4 Department of Translational Research, University of Pisa, Via Roma 67, 56126 Pisa, Italy; Dania.Cioni@med.unipi.it (D.C.); Emanuele.Neri@med.unipi.it (E.N.)

**Keywords:** CT-guided biopsy, mediastinal mass, interventional radiology, core needle biopsy

## Abstract

Purpose: To evaluate technical outcome, diagnostic yield and safety of computed tomographic fluoroscopy-guided percutaneous core needle biopsies in patients with mediastinal masses. Methods: Overall, 155 CT fluoroscopy-guided mediastinal core needle biopsies, performed from March 2010 to June 2020 were included. Size of lesion, size of needle, access path, number of success, number of biopsies per session, diagnostic yield, patient’s position, effective dose, rate of complications, tumor localization, size of tumor and histopathological diagnosis were considered. Post-interventional CT was performed, and patients observed for any complications. Complications were classified per the Society of Interventional Radiology (SIR). Results: 148 patients (age, 54.7 ± 18.2) underwent 155 CT-fluoroscopy-guided percutaneous biopsies with tumors in the anterior (114; 73.5%), middle (17; 11%) and posterior (24; 15.5%) mediastinum, of which 152 (98%) were technically successful. For placement of the biopsy needle, in 82 (52.9%) procedures a parasternal trajectory was chosen, in 36 (23.3%) a paravertebral access, in 20 (12.9%) through the lateral intercostal space and in 17 (11%) the thoracic anterior midline, respectively. A total of 136 (89.5%) of the biopsies were considered adequate for a specific histopathologic analysis. Total DLP (dose-length product) was 575.7 ± 488.8 mGy*cm. Mean lesion size was 6.0 ± 3.3 cm. Neoplastic pathology was diagnosed in 115 (75.7%) biopsies and 35 (23%) biopsy samples showed no evidence of malignancy. Minor complications were observed in 18 (11.6%) procedures and major pneumothorax requiring drainage insertion in 3 interventions (1.9%). Conclusion: CT fluoroscopy-guided percutaneous core needle biopsy of mediastinal masses is an effective and safe procedure for the initial assessment of patients with mediastinal tumors.

## 1. Introduction

Mediastinal masses include a wide spectrum of diseases, and therefore histopathological diagnosis is crucial for adequate surgical or medical treatments. For example, thymic neoplasms are primarily treated by surgery [1], whereas lymphomas or metastatic lesions are subject to a combination of chemotherapy and radiation therapy. A variety of more or less invasive methods are used for obtaining biopsies of mediastinal lesions, such as open surgical procedures, video-assisted thoracoscopic surgery (VATS), transbronchial biopsy, cervical mediastinoscopy and image-guided percutaneous needle biopsy [2]. Percutaneous image-guided core needle or Tru-Cut biopsy of mediastinal masses is usually performed under computed tomography, sonography and magnetic resonance imaging guidance, allowing detailed visualization of the lesion and the adjacent mediastinal structures as well as precise localization and documentation of the biopsy needle [3,4,5,6].

Thereby, computed tomography has been proven to be the most suitable imaging modality for core needle biopsy guidance [7]. Moreover, core needle biopsy shows higher diagnostic accuracy than fine needle aspiration, since histology is more reliable in comparison with cytology in evaluation of mediastinal lesions [5,8,9]. Furthermore, CT fluoroscopy guidance allows for real-time imaging guidance during the procedure, with fewer complications and with a lower effective patient radiation dose in contrast to sequential CT guidance [10]. Large variability regarding the diagnostic yield and complication rate of CT-guided percutaneous needle biopsy for mediastinal lesions can be found in previous studies [5,6,11,12,13,14,15].

Consequently, the purpose of this retrospective single-center study was to report the technical outcome, safety and diagnostic yield of CT fluoroscopy-guided percutaneous core needle biopsy of mediastinal masses during a 10-year period.

## 2. Materials and Methods

In this retrospective study, 148 individual patients (63 women, 85 men, 54.7 ± 18.2 (MV ± SD) years) who had undergone 155 CT fluoroscopy-guided mediastinal biopsies were included. All interventional procedures performed in our study involving human participants were in accordance with the Helsinki declaration of 1964 and with the ethical standards of the institutional and/or national research committee and its later amendments or comparable ethical standards. Regarding the review of patient charts, our institutional ethics board did not require approval of this retrospective study. All interventional procedures were performed under low-milliampere (10–20 mA) computed tomographic fluoroscopy (CTF) guidance in our institution from March 2010 to June 2020. Written informed consent from all patients was obtained a minimum of 24 h prior to the intervention after detailed explanation of the planned therapeutic intervention.

### 2.1. Study Population

Patient cohort was compiled by an RIS (radiological information system) database analysis of all CT-guided thoracic biopsies performed over a 10-year period. An interdisciplinary team of oncologists, general surgeons and interventional radiologists discussed and confirmed clinical indication for CT-fluoroscopy-guided mediastinal biopsy. This study includes only core needle or Tru-Cut biopsies (needle size ≤ 19 G) of mediastinal masses. Exclusion criteria were consistent with the general exclusion criteria for CT-guided interventions as well as the Quality Improvement Guidelines of the CIRSE and Society of Interventional Radiology (SIR) [16,17].

### 2.2. Peri-Interventional Imaging and Image Guidance

To confirm the indication of the procedure, images of CT, MRI or PET-CT, not older than two weeks, were analyzed by an experienced interventional radiologist before each intervention. Every procedure was conducted using a 16- (Somatom Sensation 16, Siemens, Healthcare GmbH, Erlangen, Germany), 64- (Somatom Sensation 64, Siemens, Healthcare GmbH, Erlangen, Germany) or 128-slice (Somatom Definition AS+; Somatom Definition Edge, Siemens, Healthcare GmbH, Erlangen, Germany) CT scanner with CT fluoroscopy (CARE Vision CT^®^, Siemens, Healthcare GmbH, Erlangen, Germany) capability. An online dose modulation system (CareDOSE 4D, Siemens Medical Solutions, Healthcare GmbH, Erlangen, Germany) was used for the pre- and post-interventional scan to adjust tube current time product (100–200 mAs) and voltage (80–120 kV). A planning CT scan was performed before the intervention to determine the anatomic localization of the lesion. In the case of a probable complex needle access path, a contrast-enhancing CT scan was acquired including a venous phase and, in some cases, an arterial phase for lesions with close contact to thoracic vessels (e.g., the internal thoracic artery). For the latter, the CT scan included 3-mm slices in axial reconstruction, as well as coronal and sagittal multiplanar reconstructions. A post-interventional CT scan was performed to visualize active bleeding or other complications. The biopsies of mediastinal lesions were performed under intermittent quick-check CT fluoroscopic acquisitions, using low-milliampere CT fluoroscopy at a tube current-exposure time product of 10 to 20 mAs. For radiation protection of the operator during intervention, thyroid shields, aprons and eyeglasses of 0.5-mm lead equivalent were applied. To reduce scattered radiation, an additional shield was put onto the lower half of the patient before sterile draping. Angular beam modulation (Hand Care^®^, Siemens, Healthcare GmbH, Erlangen, Germany) was activated during CT fluoroscopy with respect to radiation protection of the operator’s hand.

### 2.3. Procedure

All procedures were performed by one of the board-certified authors, each of them with at least 5 years of interventional experience. Monitoring with pulse oximetry was generally applied during the interventional procedure. Patients were positioned on the CT table in a prone (22/155), supine (129/155), left (2/155) or right (2/155) lateral decubitus position depending on the localization of the mediastinal mass. After choosing the diameter (16 and 18 G) and length of the Tru-Cut biopsy system (Magnum, Bard, Murray Hill, NJ, USA), sterile draping and disinfection of the skin overlying the planned needle entry point, local anesthesia with 10–20 mL of 2% mepivacaine hydrochloride (Scandicain, Astra Zeneca, London, UK) was applied. Following a small skin incision, the Tru-Cut needle was consecutively introduced under intermittent quick-check CT fluoroscopy. One to five Tru-Cut specimens were obtained by a sterilizable biopsy gun (Bard Magnum Biopsy Instrument, Covington, GA, USA). In the case of difficult handling of the relatively heavy sterilizable biopsy gun, semi-automatic disposable biopsy needles (Temno CareFusion San Diego, CA, USA or Bard Mission Biopsy Instrument, Covington, GA, USA) were used. The samples were sent to our local department of pathology for histological workup after formalin fixation. Five to ten minutes after the procedure, an unenhanced CT was obtained to examine the thorax and mediastinum, respectively, for active bleeding or other complications.

### 2.4. Assessment of Technical Outcome and Complications

Two experienced interventional radiologists evaluated the technical success and complications associated with CT fluoroscopy-guided mediastinal core needle biopsy in a retrospective by a RIS/PACS database analysis of the pre- and post-interventional CT scans, the CT fluoroscopic datasets and in the remaining medical records. The interventional procedure was defined as primarily successful if at least one histological mediastinal sample could be obtained. The cases in which a definite histopathological diagnosis of the lesion could be determined were declared as diagnostically and clinically successful. Samples including no organ tissue or regular mediastinal tissue were documented. These cases were marked as technically successful but clinically unsuccessful.

Complications of the mediastinal core needle biopsy procedures were classified according to SIR Standards of Practice Committee classification of complications by outcome [17].

### 2.5. Patient Radiation Dose

Patient radiation dose was calculated for every interventional procedure using the dose-length product (DLP (mGy*cm)), documented by the CT unit as primary dosimetric quantity data [18]. We evaluated the summarized DLP of the pre-interventional planning CT scan, the sum of all intra-interventional CT fluoroscopic acquisitions and of the post-interventional control CT scan if performed. For each intervention, effective patient radiation dose was calculated by following formula: E = DLP * t (effective dose = dose-length product * tissue weighting factor). The tissue weighting factor for the thorax region was defined as t = 0.017 [19]. This adjusted formula was utilized for evaluation of the effective dose for CT-fluoroscopy: E = DLP * k (k = 0.018) according to the computationally derived model described in detail by Leng et al. [20].

### 2.6. Statistical Analysis

The descriptive statistics are provided as percentages and counts for categorical variables, and by using the mean, standard deviation (SD), median and range for numeric variables. Discrete and continuous data were initially assessed for normality using the Shapiro–Wilk test. Based on these results variables DLP and Age are shown as the median (25%-; 75%-quartiles).

Comparison between needle diameter, number of biopsies, patient positioning, tumor entity and success of the intervention was performed with chi-square tests. Differences of DLP between two time periods and between success rate as well as between age and lesion size between success rate were assessed with Mann–Whitney-U-tests. Analysis was performed in R (R Core Team (2020)). R: A language and environment for statistical computing. R Foundation for Statistical Computing, Vienna, Austria. URL (https://www.R-project.org/, accessed on 25 April 2021) with *p* < 0.05 indicating statistical significance.

## 3. Results

### 3.1. Patient Characteristics

Patient characteristics, distribution of biopsy localization in the mediastinum and lesion size are summarized in Table 1. Mean diameter of the mediastinal lesion in the axial CT slice was 6.0 ± 3.3 (range, 0.6–18.1 cm).

### 3.2. Intervention Characteristics

One hundred forty-one patients underwent only one interventional procedure. Due to newly diagnosed lesions in the course of their disease or indefinite histopathologic results of the biopsy, seven patients underwent two interventional procedures. The interventional procedure was performed in supine position (n = 129), prone position (n = 22), right (n = 2) and left lateral position (n = 2). Access path was parasternal (n = 82), paravertebral (n = 36), in the thoracic anterior midline (n = 17) and through the lateral intercostal space (n = 20).

Mean DLP was 575.7 ± 488.8 mGy*cm, including the planning and control CT scan and the intra-interventional CT fluoroscopy images. The mean effective patient dose was 9.9 ± 8.5 mSv.

The DLP divided into two time periods (first: March 2010-mid-April 2015; second: mid-April 2015- June 2020) was significantly lower in the second time period than in the first one (*p* = 0.004). A summary of the intervention characteristics can be found in Table 2.

### 3.3. Technical Outcome and Complications

One hundred fifty-two of 155 interventional procedures (98%) were technically successful in terms of histopathological evaluation of the tissue sample. In three cases, the intervention had to be stopped without a successful biopsy because of following reasons: incompliant patients (n = 2, due to the pronounced pain symptoms/restlessness, the intervention was discontinued) and decision to cancel the biopsy due to safety reasons after evaluation of the planning CT (n = 1, access path obviated by the internal thoracic artery and vein).

One hundred thirty-six biopsies of the 152 technically successful samples resulted in definite histopathological diagnosis, consistent with clinical success (89.5%). In the remaining sixteen cases, no histopathological representative sample could be obtained. The histopathological results of the specimen obtained by CT-fluoroscopy-guided percutaneous core needle biopsy of mediastinal masses are summarized in Table 3.

A statistically significant correlation between number of biopsies and technical success rate was demonstrated. With a sample number of two, more than two and three, the technical success rate was significantly higher than in the comparison categories (*p* < 0.0001). No statistically significant associations were found between technical success rate and needle diameter, patient positioning during the interventional procedure, DLP level, patient age, lesion size, and lesion type (primary tumor vs. metastasis), respectively.

Two patients with a history of prior unsuccessful biopsy received another CT-guided biopsy, which thereupon led to a successful tissue sampling. The remaining patients with unsuccessful biopsies underwent a thoracoscopically-guided biopsy (n = 10), transbronchial (n = 1) or transoesophageal biopsy (n = 1), mediastinoscopy-guided biopsy (n = 1) and open surgical biopsy (n = 2) with successful tissue sampling, respectively.

One hundred thirty-five of 155 biopsies (87.1%) were performed without any complications. In 18 of the remaining procedures, minor complications occurred (11.6%). Fourteen of those cases were minor self-limiting hematomas along the access path. Three cases were minor pneumothoraces, evident in the post-interventional performed CT control scan. One patient exhibited a mild skin rash after contrast agent injection.

**Figure 1:**
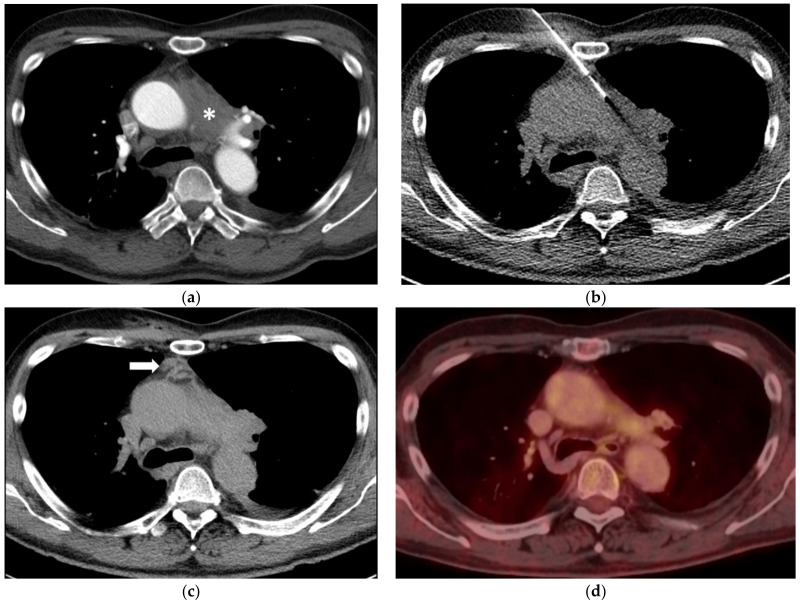
(**a**) A 65-year-old man with ill-defined paraaortic mediastinal mass (asterisk) and synchronous parailiac masses (not shown). (**b**) CT fluoroscopy-guided right parasternal access using an 18-Gauge Tru-Cut biopsy needle. The right internal mammary artery and vein are safely separated from the access trajectory by anterior mediastinal fat tissue. Histopathology reveled a chronic fibrosing mediastinitis in line with an IgG4-related disease. (**c**) Unenhanced CT after the biopsy shows a small self-limiting hemorrhage along the access path (arrow). (**d**) After six years and several doses of Rituximab, follow-up PET-CT (fusion image) confirms a stable remission with a markedly reduced paraaortic mediastinal mass (SUVmax 3.5).

**Figure 2:**
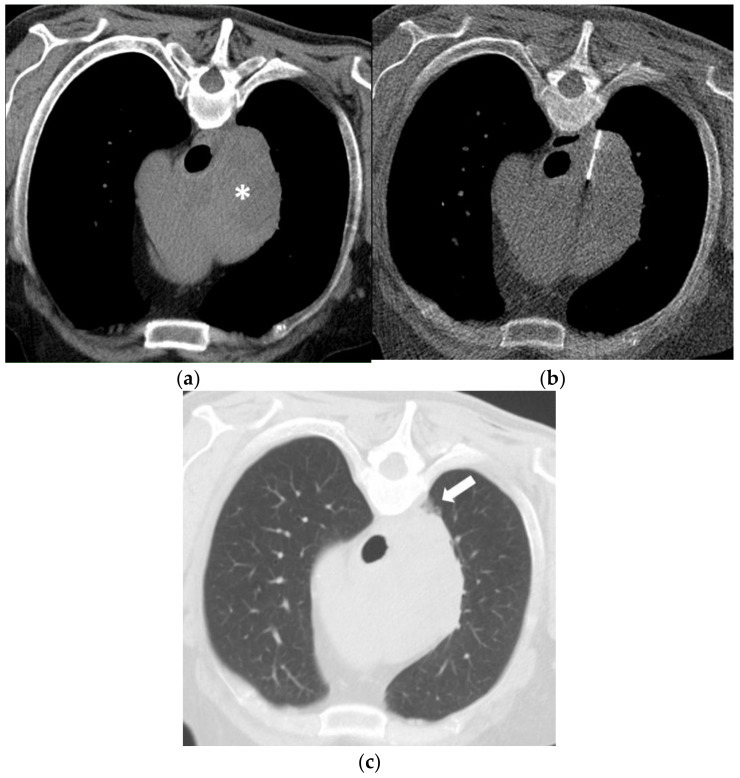
(**a**) A 60-year-old man with a history of Diffuse Large B-Cell Lymphoma (DLBCL) and missing response to CHOP chemotherapy. Unenhanced CT in prone position before biopsy shows a large right mediastinal mass (asterisk). (**b**) Using a right paravertebral transpulmonary access under CT fluoroscopy guidance, an 18-Gauge Tru-Cut biopsy needle is inserted into the mass. Histopathology confirmed a DLBCL (germinal-center-b-cell-like type). (**c**) Unenhanced CT after the biopsy depicts a small alveolar hemorrhage (arrow) without signs of a pneumothorax.

Patient A (M, 47 years, needle diameter 18 G) and patient B (M, 54 years, needle diameter 18 G): the post- interventional CT control scan revealed a small self-limiting hemorrhage along the parasternal access path (Figure 1) and a small alveolar hemorrhage after using a right paravertebral transpulmonary access (Figure 2).

**Figure 3:**
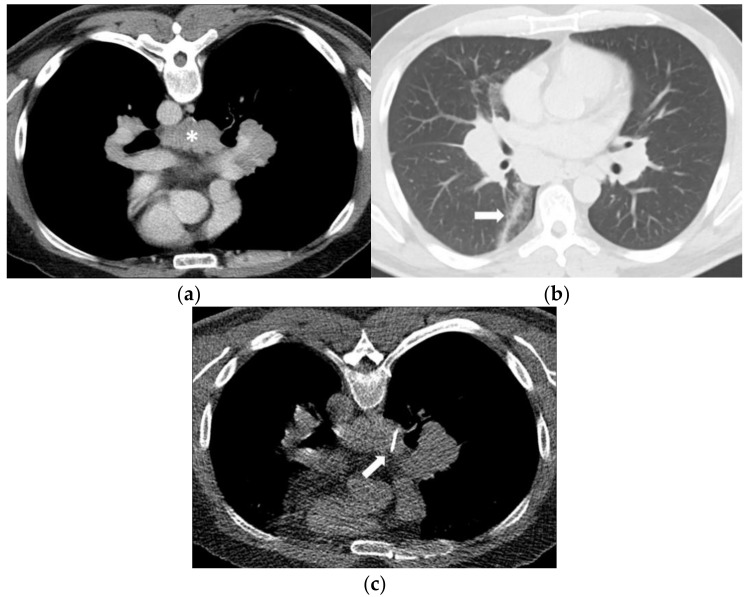
(**a**) A 48-year-old man evaluated for suspicion of lymphoma with marked mediastinal and bilateral hilar lymphadenopathy. The infracarinal mediastinal lymph node bulk (asterisk) was chosen for CT-guided biopsy after unsuccessful transbronchial fine-needle aspiration biopsy. (**b**) Using a right paravertebral transpulmonary access under CT fluoroscopy guidance, an 18-Gauge Tru- Cut biopsy needle (arrow) was inserted into the infracarinal lymph node. Histopathology revealed a granulomatous lymphadenitis in line with sarcoidosis. (**c**) Unenhanced CT after the biopsy in supine position shows a small alveolar hemorrhage along the access path (arrow) and a discrete pneumothorax.

Patient C (M, 65 years, needle diameter 18 G) underwent a CT-guided biopsy of an infracarinal lymph node. Post-interventional CT showed a small alveolar hemorrhage along the access path and a discrete self-limiting pneumothorax (Figure 3).

One patient developed a mild contrast agent allergy, which manifested in a slight skin rash. In this particular case, symptoms were controlled by anti-allergic medication.

Apart from that, none of these complications led to clinical consequences or prolonged hospitalization.

In three patients (1.9%), major complications related to the CT-guided mediastinal biopsy occurred which resulted in a major pneumothorax in all cases. In these cases, a chest tube had to be placed for therapy. No correlation was found between needle size and occurrence of complications. None of the patients had hemoptysis or major active bleeding as a complication of the biopsy.

**Figure 4:**
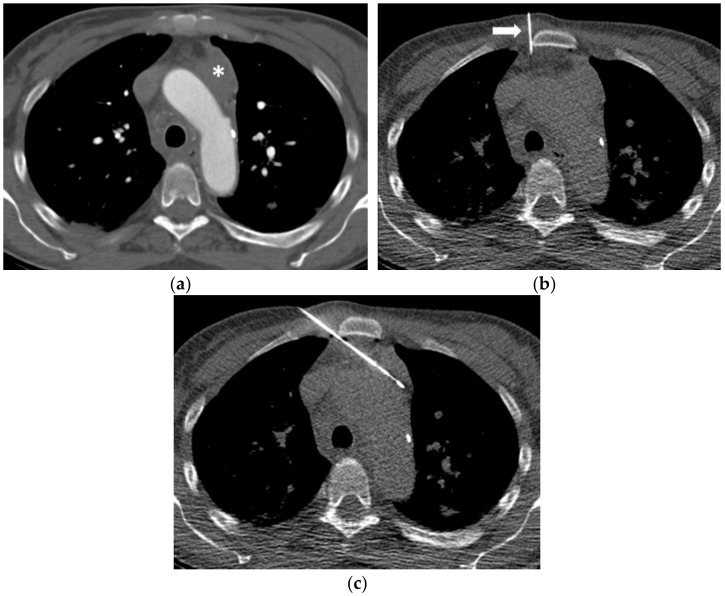
(**a**) A 48-year-old woman evaluated for suspicion of acute myeloid leukemia (AML) showing a left paraaortic mediastinal mass (asterisk). (**b**) In order to widen the parasternal soft tissue and create a safe access trajectory, 20 mL of sterile saline were injected into the parasternal fat tissue through a 22-Gauge fine needle (arrow). (**c**) Subsequently, an 18-Gauge Tru-Cut biopsy needle is safely inserted into the mass under CT fluoroscopy guidance. Histopathology confirmed a myeloid blast proliferation in line with an AML.

To avoid major complications like bleeding requiring transfusion, in several interventional procedures a safe access trajectory was created by injection of sterile saline in order to widen the space between the planned access path and the adjacent arterial vessels (Figure 4).

### 3.4. Post-Procedural Morbidity and Mortality

In the 30-day post-interventional period, no patient had complications other than those mentioned above or died attributable to the intervention. Due to rapid tumor progression, three patients died in a time interval between four to twelve weeks after the interventional procedure.

## 4. Discussion

In this study, technical outcome and diagnostic yield, as well as safety, of computed tomographic fluoroscopy-guided percutaneous core needle biopsies of mediastinal masses performed during a 10-year period at our center were investigated. A total amount of 155 procedures was evaluated which is one of the largest patient series analyzed so far; 136 interventions (89.1%) resulted in a definite histopathological result and diagnosis. Three cases were technically unsuccessful, and in sixteen procedures, no representative sample could be obtained. Most complications did not lead to further clinical consequences for the patients. In three patients, major pneumothorax occurred requiring therapy by a chest tube. With respect to technical success, diagnostic yield and complication rate associated with CT fluoroscopy-guided percutaneous core needle biopsies of mediastinal masses, our study agrees with other similar studies [3,5,6,11,12,14,21,22,23,24,25,26,27,28,29,30]. For instance, de Margerie-Mellon et al. reported a technical success rate of 100% and a diagnostic yield of 87% in 285 consecutive patients which is one of the largest studies on this subject. In this study, the overall complication rate was 7% [11]. Jiao et al. reported a complication rate of 5% [21]. Our higher complication rate (13.5%) might be explained by the fact that small hematomas along the access path without clinical relevance were reported as minor complications [16,17]. Furthermore, Jiao et al. reported on a relatively high success rate of 95.7% [26], as did Iguchi with a success rate of 97.2% [25]. Our lower success rate (89.5%) can be explained by the fact that in our study the interventional procedure was recognized as clinically successful not only if tissue was obtained but also if the specimen led to a definitive histopathologic result, reflecting the most important outcome for determining the correct treatment strategy. Remarkably, only 51 of 136 samples (37.5%) resulted in histopathological diagnosis of malignant lymphoma (Table 3), the other samples reflected a wide spectrum of metastasis, thymoma and benign lesions emphasizing the need for histological confirmation prior to starting therapy in patients with mediastinal lesions.

Comparably to a recently published meta-analysis, we were able to confirm that the diagnostic yield increases with an increasing number of biopsies (at least two to a maximum of three specimens) and when performing the interventional procedure under intermittent quick-check CT fluoroscopy [27].

Mediastinoscopy has long been one of the standard procedures for biopsy of mediastinal masses and lymph node sampling in patients with lung cancer and is particularly useful in patients who require multiple mediastinal nodes to be removed for accurate staging. It enables the direct visualization and sampling of pretracheal, paratracheal and anterior subcarinal lymph nodes and is said to provide a diagnosis in 83–89% of patients with lung cancer [4,31]. The aortopulmonary, retrotracheal, posterior subcarinal, and inferior mediastinal lymph nodes, however, are not accessible with mediastinoscopy. Mediastinoscopy also requires general anesthesia as well as an operation room and can be associated with severe complications in 1 to 3% of patients including recurrent laryngeal nerve palsy (0.05%), hemorrhage (0.32%), and tracheal injury (0.09%) [4,32]. In contrast, the rate of severe complications was 1.9% after CT-fluoroscopy-guided biopsy in our study and between 0.4 and 2.9% in other comparable studies including mostly pneumothoraces [24,25,28].

Former studies on endobronchial ultrasound-guided biopsy (EBUS) of mediastinal masses, performed during flexible bronchoscopy, reported a sensitivity for staging of non-small lung cancer of 72% and a diagnostic yield of 91% [32]. This procedure can be used to obtain fine needle aspirates (FNA) or small core samples from enlarged subcarinal and paratracheal lymph nodes. An advantage of EBUS-FNA compared to CT-guided procedures is the missing radiation exposure to patients and medical staff and a very low complication rate of 0.05% [32]. However, major limitations of this approach are the lack of accessibility to anterior lymph nodes, including those in the pretracheal and upper right paratracheal regions, due to the interposition of the air-filled trachea, as well as the need for sedation.

In our patient series, biopsy of mediastinal masses was performed under intermittent quick-check CT fluoroscopic acquisitions, using low-milliampere CT fluoroscopy (with a tube current-time product of 10 mAs) which decreases patient radiation dose and total procedure time [33,34]. The observed patient radiation exposures due to pre- and postinterventional CT as well as intra-interventional CT fluoroscopy are slightly lower than the results reported for CT drainage procedures by Kloeckner and colleagues (present study: mean DLPtotal = 575 mGy*cm; Kloeckner: mean DLPtotal = 724 mGy*cm) [18]. As stated by the authors, the use of single-slice CT fluoroscopy (in our setting the intermittent quick-check CT fluoroscopy) significantly reduces radiation exposure, while continuous (real-time) CT fluoroscopy is required only when inserting biopsy needles, drains or ablation probes for lesions that are not easily accessible [34].

In addition, the procedure must be performed by an experienced interventional radiologist (IR) to ensure a high-quality biopsy including a lower radiation dose compared to a less experienced IR which is consistent with the results of our study. In this study, up to three different experienced, board-certified interventional radiologists with at least 5 years of experience performed the intervention. A multi-professional backup team, consisting of surgery, anesthesiology and intensive care units, is also essential in the event of major complications [17].

Our analysis is characterized by several limitations. Firstly, our data are based on a retrospective single-center analysis. Secondly, we did not compare intermittent quick-check CT fluoroscopy against standard CT-guidance or continuous real time fluoroscopy, particularly with regard to radiation exposure. Finally, the heterogeneous cohort of patient’s diseases and therefore various tumor histopathologies, location of mediastinal masses and relatively small sample size are additional limitations of our study.

## 5. Conclusions

In conclusion, we have shown that CT fluoroscopy-guided core needle biopsy of mediastinal masses is effective, safe and minimally invasive and therefore represents a reliable alternative to endosonographic, transbronchial, mediastinoscopic or surgical tissue sampling. Furthermore, CT fluoroscopy-guided core needle biopsy is a complementary method after frustraneous bronchoscopic fine needle sampling. The procedure could allow a definitive histopathological diagnosis to be obtained early, thereby reducing the time to start treatment in patients with malignancies. Further prospective studies need to define the exact role and indications for CT-guided core needle biopsy compared to alternative invasive, image-guided and endoscopic methods.

## Figures and Tables

**Table 1:** Population characteristics in 148 patients having undergone CT-fluoroscopy-guided percutaneous core needle biopsy of mediastinal masses.

Variable	Numbers (Percentage)	Mean Value ± SD (Range)
Age (years)		54.7 ± 18.2 (20–89)
Sex		
Female	63 (42.6)	
Male	85 (57.4)	
Lesion size (cm)		6.0 ± 3.3 (0.6–18.1)
Mediastinal localization		
Anterior	114 (73.5)	
Middle	17 (11)	
Posterior	24 (15.5)	

**Table 2:** Characteristics and technical outcome of CT-fluoroscopy-guided percutaneous core needle biopsy of 155 mediastinal masses.

Variable	Numbers (Percentage)	Mean Value ± SD (Range)
Access path		
Parasternal	82 (52.9)	
Paravertebral	36 (23.2)	
In the thoracic anterior midline	17 (11)	
Intercostal lateral	20 (12.9)	
Needle diameter (G)		
16	33 (21.3)	
18	86 (55.5)	
Not specified	36 (23.2)	
Number of samples		
1	41 (26.5)	
2	51 (32.9)	
More than 2 *	21 (13.5)	
3	22 (14.2)	
4	3 (1.9)	
5	1 (0.6)	
Not specified	16 (10.4)	
Total effective radiation dose (Total DLP, mGy*cm)		575.7 ± 488.8 (183–3811)

* Not exactly specified in the radiologic or pathologic report.

**Table 3:** Histologic results of 152 specimens obtained by CT-fluoroscopy-guided percutaneous core needle biopsy of mediastinal masses.

Histopathology	Number	Percentage
Definite histopathologic diagnosis	136/152	89.5
Malignant lesions	115	75.7
Non-Hodgkin lymphoma	38	25
Bronchial carcinoma/metastasis	22	14.4
Hodgkin lymphoma	13	8.6
Sarcoma/metastasis	9	5.9
Malignant Thymoma	5	3.2
Extramedullary manifestation of AML/ALL/chloroma	5	3.2
Neuroendocrine tumor/metastasis	4	2.6
Breast cancer/metastasis	4	2.6
Carcinoma of unknown primary (CUP)/metastasis	4	2.6
Renal cell carcinoma/metastasis	3	2.0
Tonsil cancer/metastasis	1	0.7
Choriocarcinoma/metastasis	1	0.7
Oropharyngeal carcinoma/metastasis	1	0.7
Pancreatic carcinoma/metastasis	1	0.7
Stomach cancer/metastasis	1	0.7
Colon cancer/metastasis	1	0.7
Seminoma/metastasis	1	0.7
Non-seminomatous tumor/metastasis	1	0.7
Benign lesions	35	23
Benign Thymoma	11	7.2
Hematoma	7	4.5
Pericardial cyst/effusion	4	2.6
Thymus	4	2.6
Mediastinal inflammatory pseudotumor	2	1.3
Lymphadenitis	2	1.3
Lipoma	1	0.7
Struma	1	0.7
Sclerosing mediastinitis	1	0.7
Scar tissue	1	0.7
Schwannoma	1	0.7
Miscellaneous	2	1.3
Teratoma	2	1.3

## Data Availability

The data presented in this study are available upon reasonable request from the corresponding author.
